# Development of experimental ground truth and quantification of intracranial aneurysm pulsation in a patient

**DOI:** 10.1038/s41598-021-88420-3

**Published:** 2021-05-03

**Authors:** Axel E. Vanrossomme, Kamil J. Chodzyński, Omer F. Eker, Karim Zouaoui Boudjeltia

**Affiliations:** 1 Laboratory of Experimental Medecine (ULB 222 Unit), Medicine Faculty, Université Libre de Bruxelles, CHU de Charleroi, 6110 Montigny-le-Tilleul, Belgium; 2 Medical Imaging Unit, Centre Hospitalier Universitaire de Charleroi, 6042 Charleroi, Belgium; 3 Hospices Civils de Lyon, Neurointerventional Radiology, 69003 Lyon, France

**Keywords:** Aneurysm, Computed tomography

## Abstract

Aneurysm wall motion has been reported to be associated with rupture. However, its quantification with medical imaging is challenging and should be based on experimental ground-truth to avoid misinterpretation of results. In this work a time-resolved CT angiography (4D-CTA) acquisition protocol is proposed to detect the pulsation of intracranial aneurysms with a low radiation dose. To acquire ground-truth data, the accuracy of volume pulsation detection and quantification in a silicone phantom was assessed by applying pressure sinusoidal waves of increasing amplitudes. These experiments were carried out using a test bench that could reproduce pulsatile waveforms similar to those inside the internal carotid arteries of human subjects. 4D-CTA acquisition parameters (mAs, kVp) were then selected to achieve reliable pulsation detection and quantification with the lowest radiation dose achievable. The resulting acquisition protocol was then used to image an anterior communicating artery aneurysm in a human subject. Data reveals that in a simplified in vitro setting 4D-CTA allows for an effective and reproducible method to detect and quantify aneurysm volume pulsation with an inferior limit as low as 3 mm^3^ and a background noise of 0.5–1 mm^3^. Aneurysm pulsation can be detected in vivo with a radiation dose approximating 1 mSv.

## Introduction

Endovascular and surgical treatments of unruptured intracranial aneurysms are associated with substantial morbidity and mortality^1–3^. As the potential benefits of these treatments should overweigh their risks, the assessment of the rupture probability should be as accurate as possible. Currently, most clinicians base their decision to treat on the Unruptured Cerebral Aneurysm Study (UCAS) and the PHASES score^4,5^ but additional criteria could help to identify aneurysms that are prompt to rupture. Aneurysm pulsation, also called ‘wall motion’, could be one of these criteria as its possible association with rupture has been suggested^6–9^. Aneurysm pulsation can be detected and quantified by various imaging techniques: transcranial power Doppler ultrasonography (PD-US); phase-contrast magnetic resonance angiography (PC-MRA); dynamic computed tomographic angiography (4D-CTA); and three-dimensional rotational angiography (3D-RA)^7,9–13^. Among these techniques, PD-US and PC-MRA have the lowest spatial resolutions and are the most susceptible to flow artifacts^12,14^ while 3D-RA is the most invasive. 4D-CTA could therefore be the best compromise between spatial resolution and invasiveness despite the need for ionizing radiation^15^. Numerous acquisition parameters have been reported with wide ranges of time–current products (60–270 mAs) and of tube potentials (80–120 kV) therefore inducing wide ranges of radiation doses^11,16^. Additionally, experimental ground-truth was stated to be important to avoid pitfalls in the interpretation of in vivo imaging studies^17^. The purpose of this work was therefore to propose a 4D-CTA acquisition protocol to best detect intracranial aneurysm volume pulsation pulsation at the lowest achievable radiation dose in patients based on in vitro experimental ground-truth.

## Methods

**Figure 1:**
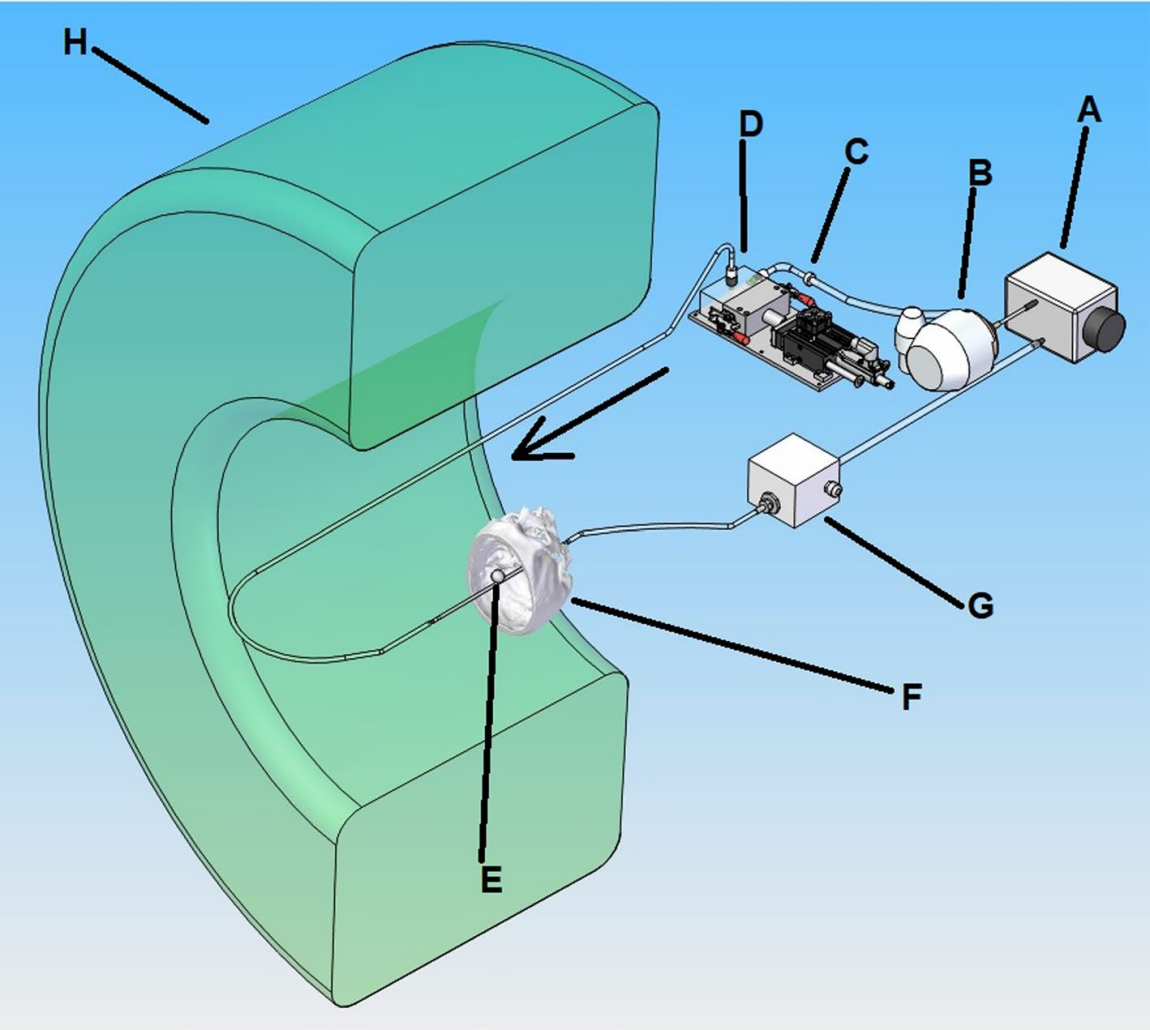
Hydraulic circuit of the in vitro test bench. (**A**) reservoir; (**B**) centrifugal pump; (**C**) check valve; (**D**) piston pump that consists in a linear motor and a piston with a diaphragm; (**E**) examined aneurysm model; (**F**) partial human skull; (**G**) electromagnetic flowmeter. Figure made by KJ Chodzyński using SolidWorks 2018 software (Dassault Systèmes, Vélizy-Villacoublay, France).

This study received approval from two ethics committees (*Comité d’éthique Erasme-ULB* and *Comité d’éthique du CHU de Charleroi—ISPPC*) for data collection and use of a non-routine CT scan in patients. All experiments were performed in accordance with relevant guidelines and regulations. As a first step, we determined the smallest detectable pulsation in silicone phantoms mimicking aneurysms. As a second step, we determined the 4D-CTA tube parameters (mAs, kVp) able to detect this pulsation and recorded the radiation dose associated with these parameters. These two steps were conducted in vitro using a test bench simulating blood flow (European patent reference EP2779144—Fig. 1)^18^. As a third step, we tested the acquisition parameters by verifying that the pulsation was detectable in vivo in a patient. All acquisitions were made on a 64 detector-rows CT scanner (Somatom Definition AS, Siemens Healthineers, Erlangen, Germany) with z-flying focal spot X-ray tube (Straton, Siemens).

### Step 1: pulsation detection

A test bench was used to apply reproducible pulsatile flow inside a silicon aneurysm phantom. The circuit was filled with a solution of glycerin and water that was close to the average human blood viscosity (3.45 × 10^–3^ Pa⋅s at 20 °C). This solution was mixed with Iopromide 370 mgI/ml (Ultravist 370, Bayer HealthCare, Leverkusen, Germany) at a 4:1 volume ratio to achieve appropriate opacification. The centrifugal pump was set up to generate a mean flow of 180 ml/min. The piston pump frequency was set at 1.5 Hz and the pulsation amplitude was gradually increased to fit the desired pulse pressure that was recorded concurrently. The mean pressure was set at 90 mmHg by means of valves. A spherical silicon aneurysm phantom was connected to the test bench and placed inside a partial human skull filled with ultrasound transmission gel (Aquasonic 100, Parker Laboratories, Fairfield, NJ) to mimic the X-ray attenuation of the brain and the cerebrospinal fluid. The parameters for stationary acquisitions were as follows: peak kilovoltage = 120 kVp; time–current product = 190 mAs; acquisition scan time = 5 s; tube rotation time = 0.3 s. A volume with a thickness of 3.84 cm (64 slices) was then reconstructed with a 150 ms time interval. This whole process was performed three times on separate days with the experimental setup made anew to account for unexpected variations. Image post-processing and analysis was performed as described below. Pulsation was defined as the difference between zenith and nadir of each pulse (7 pulses per experiment). Average volume pulsation, standard deviations and coefficients of variation were then calculated for each experiment. In addition, we analyzed the correlation between volume pulsation and pulse pressure.

### Step 2: In vitro optimization of CT parameters

The same test bench, scanner device, phantom, skull and contrast medium as described above were used. The piston pump was set at a frequency of 1.5 Hz to generate a pulse pressure of approximately 200 mmHg. The following CT parameters were used: scan duration = 5 s; tube potential = 80 and 100 kV; time–current product = 70, 110, 150, and 190 mAs for each value of tube potential.

We verified that the radiation dose displayed on the CT screen after acquisition was accurate by comparing this dose to that measured with an ionization chamber inserted in a 16 cm Plexiglas phantom. The difference was no larger than 3%, in agreement with the legal requirements^19^.

Each acquisition was performed three times on separate days with the experimental setup made anew to account for unexpected variations. Average volume pulsation, standard deviations and coefficients of variation were then calculated for each experiment.

We also recorded the radiation doses for the acquisition parameters used in step 2, expressed through volume CT dose index (CTDI_vol_) and dose length product (DLP). The dose to the skin was estimated by the CTDI_100_ (dose distribution over a 100 mm long ionization chamber) which equals CTDI_vol_ × conversion factor of 1 for head CT acquisition. The effective dose was calculated by multiplying the DLP by the head region-based conversion factor of 0.0023 mSv/(mGy⋅cm)^20^.

### Step 3: In vivo validation in a patient

Inclusion criteria were as follows: at least one intracranial aneurysm larger than 7 mm in diameter, not in intrapetrous or intracavernous location; more than 18 years of age; not pregnant; no renal or cardiac failure or any history of allergic-like reaction related to iodinated contrast agent. One patient bearing a 9 mm anterior communicating artery saccular aneurysm was included after receiving their written informed consent. The aneurysm was imaged by using the CT acquisition parameters determined in vitro as described above. To ensure adequate timing of acquisition a bolus tracking method was used. A slice passing through the thoracic aorta was scanned repeatedly 10 s after iodine contrast injection (Iopromide 370 mgI/ml, 100 ml at a rate of 4 ml/s, saline flush with 60 ml of a solution of NaCl 9‰), until mean attenuation inside a ROI placed in the descending aorta was > 100 UH. The 5 s-long dynamic acquisition then started after a fixed 10 s delay. The patient’s electrocardiogram was recorded during the acquisition and synchronized with the scanner clock. Reconstruction method was similar to that used in vitro except for a reduced time increment of 50 ms in order to avoid suboptimal sampling observed in vitro as a source of error (see Results section). Each time step volume value was represented graphically and pulsation was assessed visually based on resulting graph of aneurysm volume as a function of time, by comparing the pulsatile pattern to the electrocardiogram. Pulsation was calculated as the difference between the zenith of each pulse and the mean of both adjacent nadirs to take into account the variation of apparent volume related to inconstant iodine contrast concentration inside the vascular lumen.

### Image post-processing and analysis

The post-processing and image analysis for the first, second, and fourth steps were carried out as follows. Pulsation analysis was performed using MATLAB 2015b software (MathWorks, Natick, MA) with additional toolboxes (Parallel Computing Toolbox 6.7 and Image processing Toolbox 9.3). 2D DICOM images were organized as a 3D matrix (X, Y, Z) where X and Y units were the pixel size of the 2D DICOM image and Z unit was the slice thickness. However, X–Y pixel size being smaller then slice thickness, the 3D matrix was further subjected to a linear interpolation using ‘Imwarp’ MATLAB function to obtain isotropic voxels with nominal size equal to that of the x–y pixels. This grey scale matrix was then converted to a black-white matrix. A Hounsfield Unit (HU) threshold was set by visual assessment to choose the threshold that best fitted the aneurysm contours. All voxels with attenuation coefficient lower than that threshold were given a value of 0 whereas all voxels with attenuation coefficient higher than that threshold were given a value of 1 hence resulting in a black-white 3D matrix. Masks were applied to the resulting 3D matrix. This post-processing was done differently for the in vitro study and for that in patients. The simplified in vitro settings allowed us to limit to two masks. The first mask was added to decrease the time calculation cost as well as to remove the skull bones by extracting a region of interest (ROI) using ‘imcrop’ MATLAB function which covered only the ROI containing the aneurysm. The second mask was a ‘XY’ elliptic mask applied manually on the vessel by using ‘imellipse’ MATLAB function to remove the parent vessel. However, as the vessel was not perfectly straight, an algorithm that followed the position of the vessel on each 2D slice was applied. As a result, the aneurysm sac was segmented. For the in vivo study the masks were applied differently. As the patient aneurysm can indeed have various shapes and conformations, it was necessary to apply masks on multiple planes (XY, XZ, YZ). The ‘impoly’ MATLAB function creating polygonal masks was therefore used. By applying those masks it was possible to extract only the aneurysm sac. To calculate the volume of the aneurysm sac, the following equation was used.

Volume (mm^3^) = N_w_ ⋅ X_v_ ⋅ Y_v_ ⋅ Z_v_ with

- N_w_ = the total number of white voxels;
- X_v_ = size of the voxel in the X direction [mm];
- Y_v_ = size of the voxel in the Y direction [mm];
- Z_v_ = size of the voxel in the Z direction [mm]

The whole process was repeated automatically for all time steps.

## Results

### Step 1: pulsation detection

**Figure 2:**
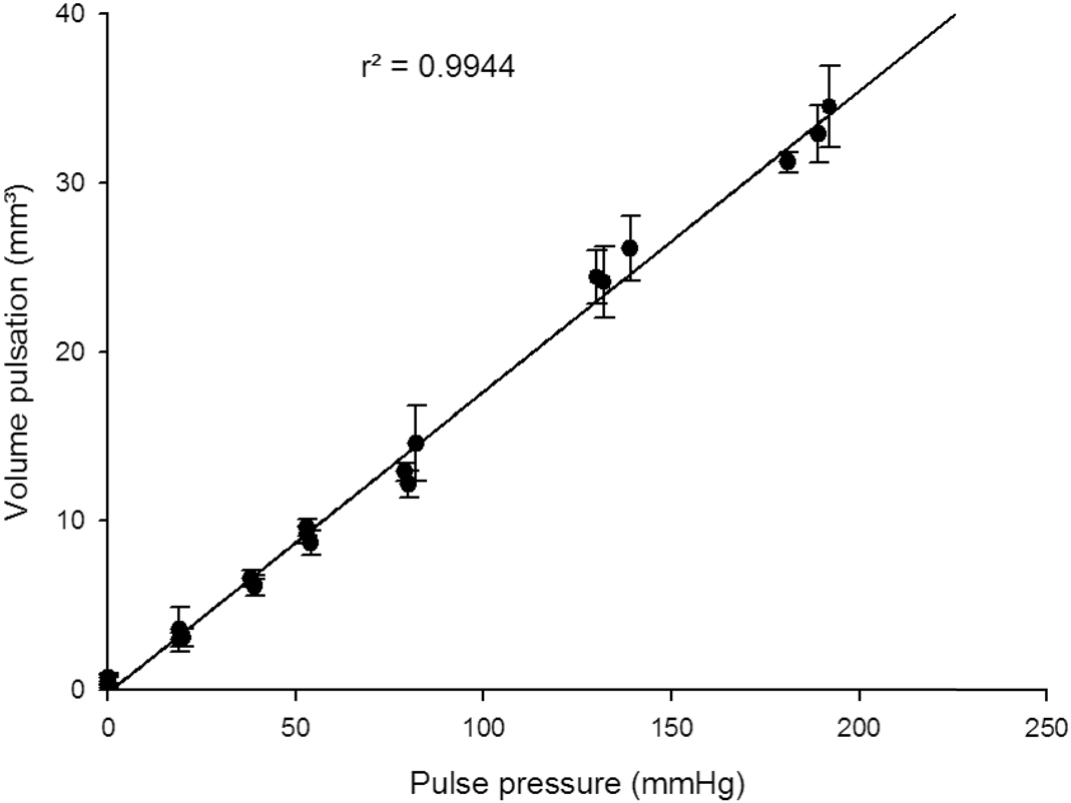
Volume pulsation as a function of pulse pressure (in vitro setting) with regression line and correlation coefficient. Error bars represent standard deviation.

**Figure 3:**
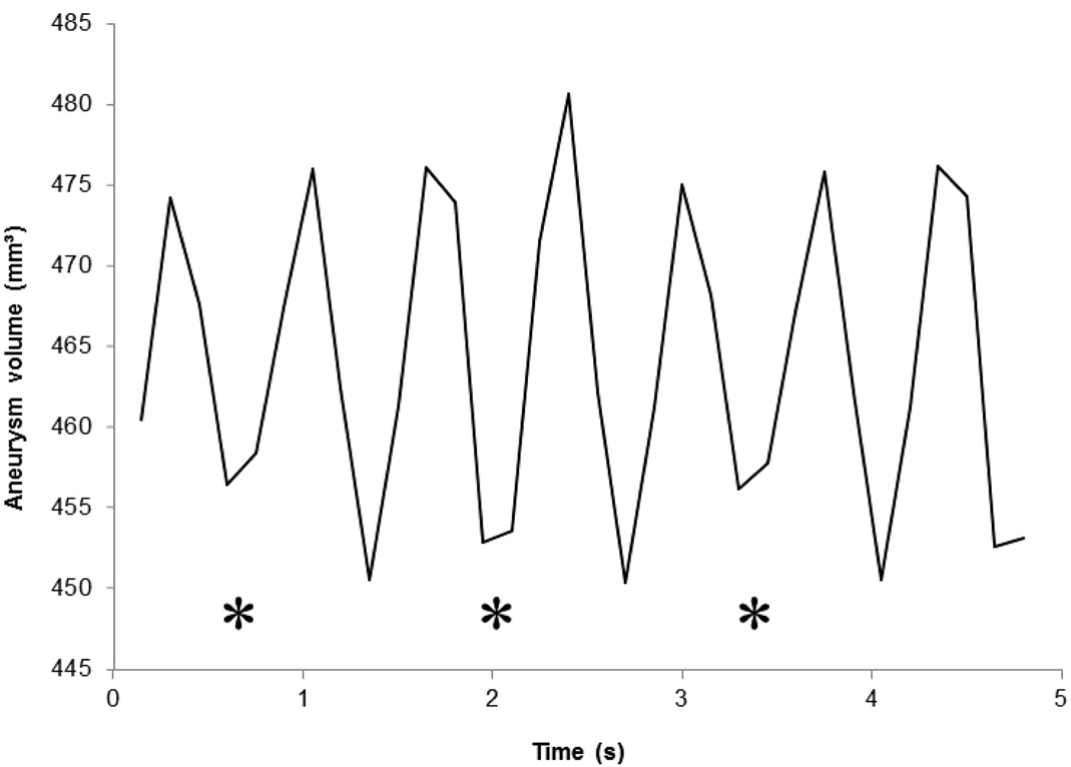
Example of suboptimal temporal sampling (in vitro setting). Nadirs marked with a star (*) are truncated.

Average volume pulsation and standard deviation are detailed for each experiment in Table 1. As expected, the quantified volume pulsation increased with increasing pulse pressure. Additionally, the relation between volume pulsation and pulse pressure was linear within the range of tested pulse pressures with a high determination coefficient (r^2^ > 0.99) (Fig. 2). When the pulse pressure was set at 0 mmHg, an artefactual pulsation appeared but its period was different than that of the pump and variance in amplitude was higher than with any other setup. This defined the background noise of the technique at 0.5–1 mm^3^. The minimal pulsation that could be unequivocally distinguished from the noise was 3 mm^3^. Coefficients of variation ranged from 0.03 to 0.35 for experiments at non-null pulse pressure, most of them being between 0.05 and 0.10 when pulse pressure was ≥ 40 mmHg. It is worth mentioning that most of the variation appeared to be a result of suboptimal temporal sampling (Fig. 3) rather than related to other CT parameters.

**Table 1:** Aneurysm model pulsation with increasing pulse pressure.

Experiment	PP (mmHg)	Number of pulsations	AVP ± SD (mm^3^)	CoV (%)
0.1	0	7 (artefactual)	0.7 ± 0.3	43
0.2	0	8 (artefactual)	0.5 ± 0.3	53
0.3	0	8 (artefactual)	0.6 ± 0.3	42
1.1	19	7	3.6 ± 1.3	35
1.2	20	7	3.1 ± 0.5	15
1.3	19	7	3.0 ± 0.4	12
2.1	39	7	6.1 ± 0.5	9
2.2	38	7	6.6 ± 0.5	7
2.3	39	7	6.2 ± 0.6	10
3.1	54	7	8.7 ± 0.7	8
3.2	53	7	9.6 ± 0.5	5
3.3	53	7	9.2 ± 0.5	5
4.1	80	7	12.2 ± 0.8	7
4.2	82	7	14.6 ± 2.2	15
4.3	79	7	12.9 ± 0.5	4
5.1	132	7	24.1 ± 2.1	9
5.2	139	7	26.1 ± 1.9	7
5.3	130	7	24.4 ± 1.6	7
6.1	189	7	32.9 ± 1.7	5
6.2	192	7	34.5 ± 2.4	5
6.3	181	7	31.2 ± 0.6	2

PP: pulse pressure; AVP: average volume pulsation; SD: standard deviation; CoV: coefficient of variation. Experiment first digit refers to each experimental setup, the second digit, after the dot, refers to each repetition.

### Step 2: In vitro optimization of CT parameters

Average volume pulsation with standard deviation and coefficient of variation are detailed for each experiment in Table 2. The radiation doses delivered by each CT protocol are summarized in Table 3. They ranged from 0.45 to 2.43 mSv.

**Table 2:** Aneurysm model volume pulsation with various acquisition parameters.

Experiment number	Tube potential (kV)	Time–current product (mAs)	Number of pulsations	AVP ± SD (mm^3^)	CoV (%)	D_eff_(mSv)
1.1	80	70	7	26.0 ± 3.3	13	0.4508
1.2	80	70	7	26.1 ± 1.1	4	0.4508
1.3	80	70	7	24.4 ± 3.7	15	0.4508
2.1	80	110	7	24.7 ± 1.8	7	0.7015
2.2	80	110	7	23.0 ± 3.1	13	0.7015
2.3	80	110	7	23.4 ± 2.7	11	0.7015
3.1	80	150	7	25.0 ± 1.2	5	0.9591
3.2	80	150	7	23.5 ± 4.2	18	0.9591
3.3	80	150	7	23.6 ± 2.2	9	0.9591
4.1	80	190	7	23.3 ± 2.4	10	1.2121
4.2	80	190	7	22.8 ± 2.2	10	1.2121
4.3	80	190	7	24.1 ± 2.9	12	1.2121
5.1	100	70	7	20.5 ± 2.9	14	0.8993
5.2	100	70	7	24.2 ± 4.0	16	0.8993
5.3	100	70	7	23.1 ± 3.8	16	0.8993
6.1	100	110	7	22.8 ± 2.4	11	1.4053
6.2	100	110	7	20.7 ± 1.4	7	1.4053
6.3	100	110	7	21.7 ± 2.4	11	1.4053
7.1	100	150	7	22.1 ± 1.0	5	1.9205
7.2	100	150	7	22.6 ± 2.8	13	1.9205
7.3	100	150	7	22.6 ± 2.6	12	1.9205
8.1	100	190	7	19.7 ± 3.2	16	2.4311
8.2	100	190	7	22.6 ± 1.3	6	2.4311
8.3	100	190	7	21.2 ± 1.1	5	2.4311

AVP: average pulsation volume; SD: standard deviation; CoV: coefficient of variation; D_eff_: effective dose. Experiment first digit refers to each experimental setup, the second digit, after the dot, refers to each repetition.

**Table 3:** Radiation dose associated with each acquisition protocol.

Tube potential (kV)	Time–current product (mAs)	Scan Time (s)	CTDI_vol_ (mGy)	CTDI_100_ (mGy)	DLP (mGy.cm)	D_eff_ (mSv)
80	70	5	50.93	50.93	196	0.45
80	110	5	79.44	79.44	305	0.70
80	150	5	108.60	108.60	417	0.96
80	190	5	137.33	137.33	527	1.21
100	70	5	101.93	101.93	391	0.90
100	110	5	158.99	158.99	611	1.41
100	150	5	217.37	217.37	835	1.92
100	190	5	275.30	275.30	1057	2.43

CTDI_vol_: volume computed tomography dose index; CTDI_100_: computed tomography dose index over a 100 mm long ionization chamber; DLP: dose-length product; D_eff_: effective dose.

**Figure 4:**
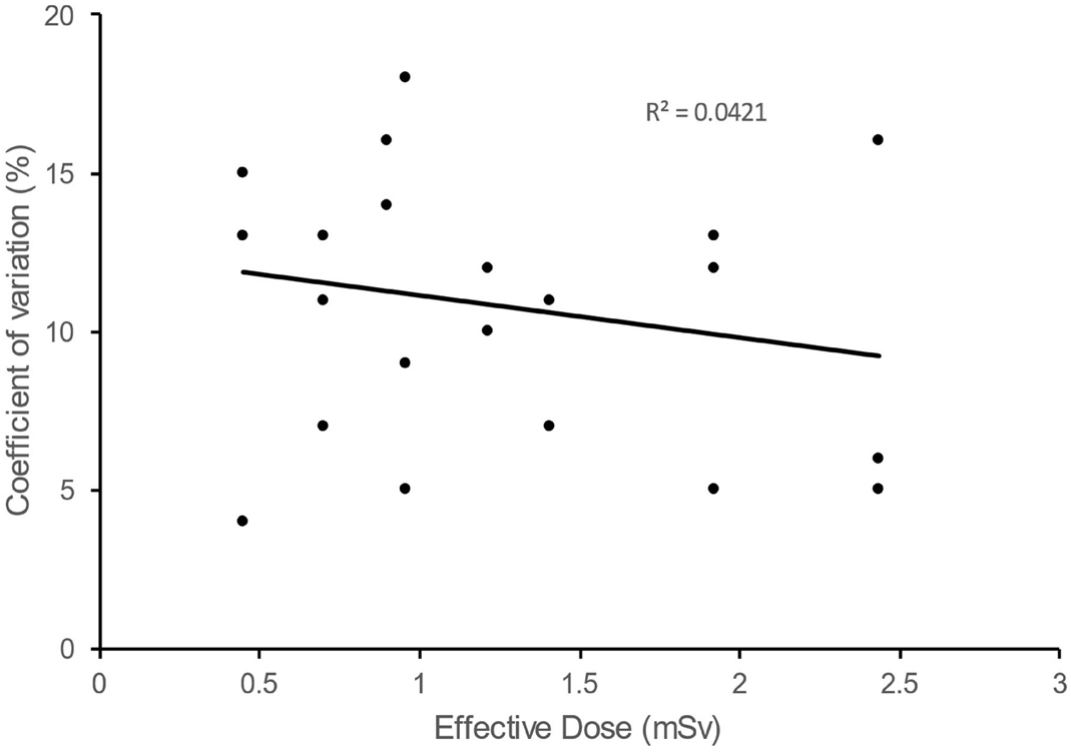
Coefficient of variation as a function of effective dose (in vitro setting) with regression line and correlation coefficient.

There was very little decrease in coefficient of variation with increasing radiation dose (Fig. 4).

### Step 3: In vivo validation in a patient

**Figure 5:**
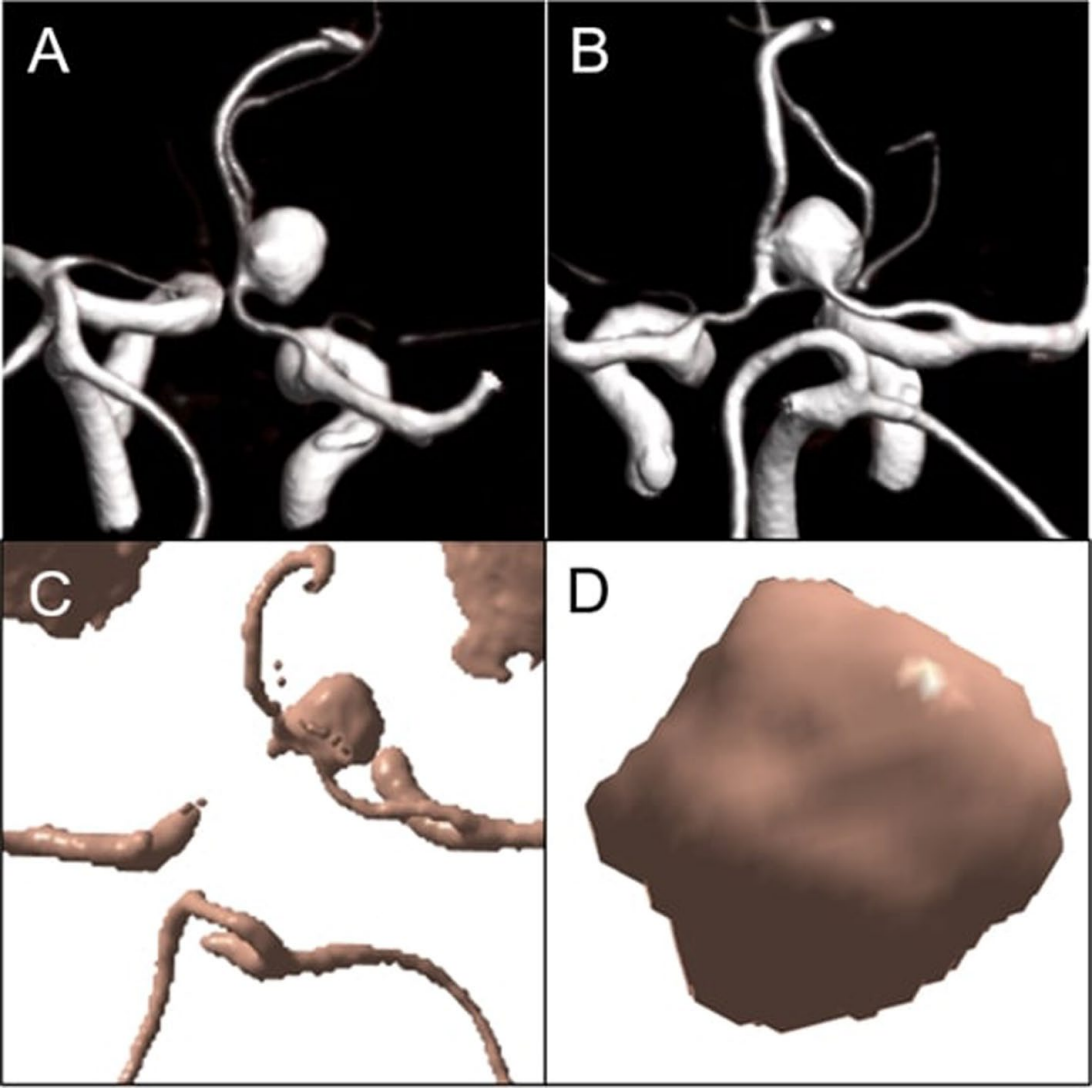
CT-angiography reconstructions of the anterior communicating artery aneurysm (**A**, **B**). Post-processing of the 4D-CTA scan after application of a Hounsfield Unit threshold (**C**) and after application of masks (**D**). Images **A** and **B** obtained using Syngo.Via VB30 software (Siemens Healthineers, Erlangen, Germany) and images **C** and **D** using MATLAB 2015b software (MathWorks, Natick, MA).

**Figure 6:**
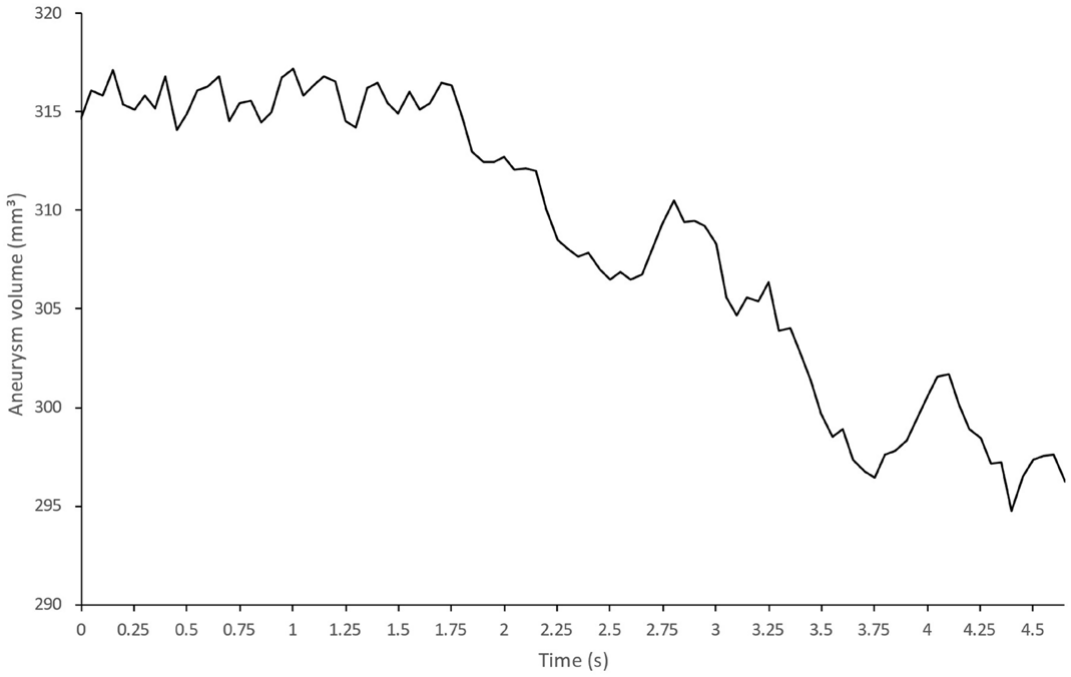
Apparent aneurysm volume over time (in vivo).

**Figure 7:**
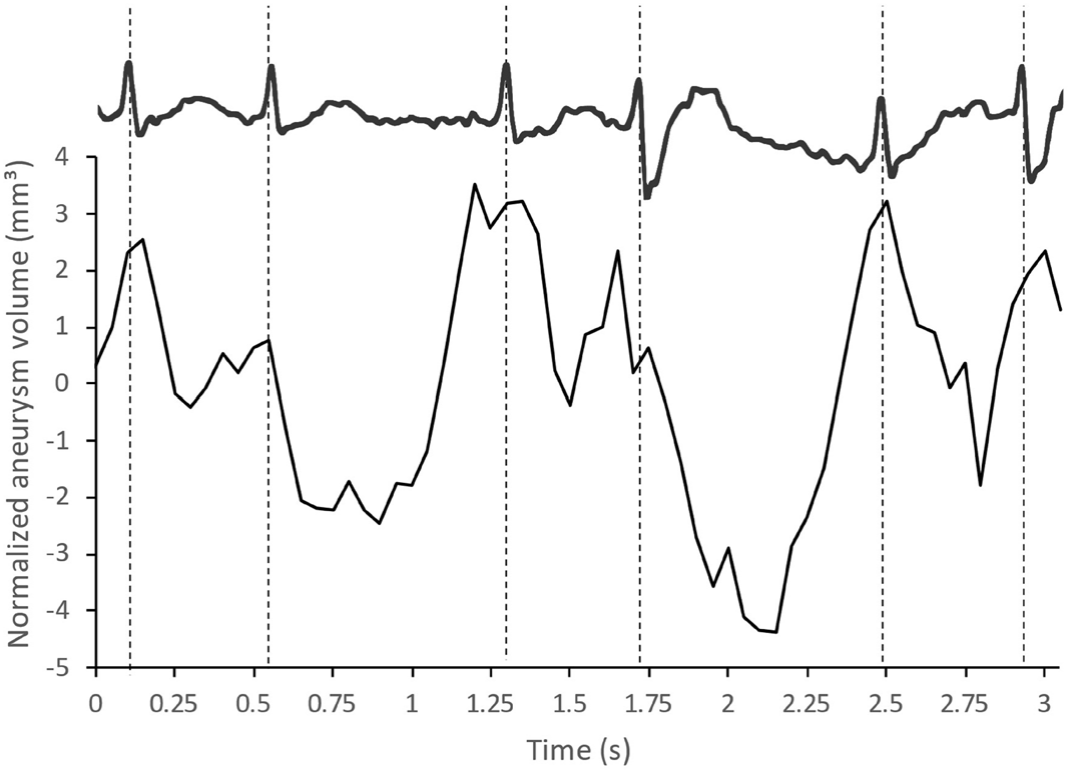
Aneurysm volume over time and relation to ECG (in vivo). Normalized volume defined as the difference between volume at each timepoint and the average volume defined by the linear interpolation of the downward slope of the graph in Fig. 6.

The included patient was a 58 years old woman who presented with a fortuitously discovered unruptured aneurysm located on the anterior communicating artery (Fig. 5). The largest aneurysm diameter measured 9 mm for a volume of approximately 310 mm^3^. The graph of aneurysm volume over time (Fig. 6) shows an initial segment where some fluctuation occurs but no clear pulsation can be detected, followed by a downward slope where five pulsations are detected that closely match electrical heart activity at the time of acquisition. This segment of the graph where pulsations were more apparent was subsequently normalized to suppress the effect of the decrease in contrast concentration over time (Fig. 7). Pulsation amplitude ranged from 2.7 to 6.1 mm^3^ (average: 4.7 mm^3^—standard deviation: 1.5 mm^3^). The 4D-CTA acquisition delivered a radiation dose of 0.95 mSv (CTDI_vol_: 108.2 mGy; DLP: 415 mGy⋅cm). To this dose must be added the ones from the topogram and the bolus tracking, thus resulting in a total radiation dose of 1.03 mSv (DLP: 446 mGy⋅cm). A video of the dynamic angio-scanner can be found in supplementary information (Supplementary Video S1).

## Discussion

In optimal in vitro conditions, 4D-CTA reliably detects aneurysm pulsation. Quantification is reproducible but the lack of reference technique makes it impossible to affirm that it is accurate. However, our results showed a high correlation between the detected amplitude of pulsation and the amplitude of pulse pressure which suggests that quantification is indeed accurate, or at the very least, relevant. There was some variance in pulsation quantification but part of the observed variance could be explained by suboptimal temporal sampling (150 ms time interval between two successive reconstructions). Temporal resolution was increased up to 50 ms on later generations of scanners which most probably would lead to a decrease in variance. This lower time increment came with the caveat that each consecutive reconstruction would not contain unique data as 180° of projections are required for one volume reconstruction (150 ms for a rotation time of 300 ms in our setting) though it seemed to be outweighed by the potential advantages. This higher temporal resolution was therefore implemented for the study on patients.

Unexpectedly, there was very little decrease in coefficient of variation with increasing radiation dose. which would suggest that the acquisition parameters with the lowest dose would be the most appropriate. However, we chose to use other acquisition parameters (tube potential of 80 kV and time–current product of 150 mAs) that delivered a higher dose because we expected image processing and volume quantification to be more difficult in vivo than in vitro.

Our results show that the in vivo detection of aneurysm pulsation is feasible. There are however numerous issues that need to be resolved. Firstly, the optimal timing of acquisition is difficult to accomplish. It is theoretically better to image the aneurysm when iodine concentration is constant within its lumen. However this cannot be reliably achieved because the shape of the curve of contrast concentration according to time depends on multiple factors such as the volume of contrast injected, the rate of injection and the cardiac function of the patient, even through the use of bolus tracking techniques. This technical point might be improved in the future. Secondly, image post-processing and analysis were based on a Hounsfield Unit threshold which is imprecise. Small variations of contrast concentration could therefore result in artefactual volume variations impairing pulsation detection and quantification. A recent approach based on adaptive thresholding could help improve pulsation detection and quantification^21^. Additionally, global arterial motion during cardiac cycle could also cause artefactual pulsation through partial volume effect or segmentation variations. Further improvements in post-processing could focus on correcting for this motion. Thirdly, although aneurysm pulsation detection has been reported without electrocardiogram (ECG) recording during acquisition, it greatly helped during the analysis process and increased confidence in the validity of the results. However, it required the use of a separate monitoring device synchronized with the clock of the CT-scanner because the ECG could not be recorded on our CT-scanner while using a non-gated time-resolved acquisition. Such an integrated acquisition technique would facilitate the exam procedure and the post-processing.

Pulsation was detected on a 9 mm aneurysm and pulsation amplitude was on the low range of detectability. Therefore it would probably be challenging to detect pulsation in smaller aneurysms. Interestingly, “softer” wall biomechanical properties were reported in preruptured and ruptured aneurysms^22^. Pulsation could be increased in such aneurysms and detectable in smaller ones making this a potentially tool to anticipate rupture in smaller aneurysms^15^. Moreover, had experimental ground-truth not been available, interpretation of results would have been much more challenging. Artefactual volume fluctuation that was apparent on the initial part of the graph of volume over time (Fig. 4) could have been interpreted as actual pulsation if the limit of detection of the technique was unknown.

The radiation dose delivered by the time-resolved acquisition approximated 1.0 mSv (dose-length product of 425 mGy⋅cm) which is inferior to the 25th percentile of the reference dose levels for head CT scans (640 mGy⋅cm) as published by the Belgian Federal Agency for Nuclear Control^23^ and to the U.S. achievable dose (50th percentile) for head CT scans^24^. Delivered radiation dose was rarely reported in studies of aneurysm pulsation using 4D-CTA. This information was only found in two studies by Firouzian et al. and Krings et al. that respectively reported radiation doses of 0.9–1.1 mSv and of 3.592 mSv^11,25^. The first one used a helical CT acquisition with ECG-gating on a 32 detector-rows CT scanner and necessitated a CT angiography to locate the aneurysm. Another author has however reported that a 4D-CTA acquired with no table movement had a higher rate of aneurysm pulsation detection than helical 4D-CTA^26^. The second one used an acquisition with no table movement on a 320 detector rows CT scanner. However, a 16 cm scan length is probably not needed for most aneurysms and the acquisitions can usually be correctly planned using bony landmarks and former imaging studies. Therefore, 4 to 6 cm scan length are sufficient which in turn lowers the radiation dose.

This study has several limitations: (1) the detection and quantification of aneurysmal pulsation is a recent subject of interest, therefore there is currently no reference technique for aneurysm pulsation measurement; (2) validation is based on a single patient but the purpose was to verify that pulsation could indeed be detected; (3) the post-processing and analysis techniques are still flawed and could be improved. Different potential solutions have been discussed above.

In conclusion, this study shows that: (1) in a simplified in vitro setting 4D-CTA allows for an effective and reproducible way to detect and quantify aneurysm pulsation with an inferior limit of 3 mm^3^ below which pulsation cannot be reliably detected; (2) aneurysm pulsation can be detected in vivo but experimental ground-truth is critical to avoid misinterpretation of results; (3) such detection can be achieved with a radiation dose approximating 1 mSv.

Whether this technique can be applied in clinical practice to anticipate rupture in aneurysms that would not otherwise require treatment should be prospectively evaluated.

## Supplementary Information

Supplementary Video 1.

Supplementary Information 1.
