# Supplementary material for: Development of experimental ground truth and quantification of intracranial aneurysm pulsation in a patient
Source: Sci Rep. 2021 May 3;11:9441. doi: 10.1038/s41598-021-88420-3 (PMC8093229; doi:10.1038/s41598-021-88420-3)
Supplement: Supplementary file 2 — Supplementary Information 1. [file 41598_2021_88420_MOESM2_ESM.docx]

**Development of experimental ground truth and quantification of intracranial aneurysm pulsation in a patient**

**Supplementary Information**

**Supplementary Video S1.** Video of the dynamic angio-scanner of the aneurysm showing a slight motion of the wall in the inferior half of the sac. Video recorded Syngo.Via VB30 software (Siemens Healthineers, Erlangen, Germany)
